# Characteristics of a population of commuter cyclists in the Netherlands: perceived barriers and facilitators in the personal, social and physical environment

**DOI:** 10.1186/1479-5868-7-89

**Published:** 2010-12-10

**Authors:** Luuk H Engbers, Ingrid JM Hendriksen

**Affiliations:** 1 TNO Quality of Life, Leiden, The Netherlands; 2 Body@Work, Research Center Physical Activity, Work and Health, TNO-VUmc, Amsterdam, The Netherlands

## Abstract

**Background:**

Daily cycling to work has been shown to improve physical performance and health in men and women. It is very common in the Netherlands: the most recent data show that one quarter of commuting journeys are by bicycle. However, despite the effort going into campaigns to promote commuter cycling, about 30% of commuter journeys up to 5 kilometers are still by car. The question is how to stimulate commuter cycling more effectively. This article aims to contribute to a better understanding of the perceived barriers and facilitators of cyclists/non-cyclists and personal factors associated with commuter cycling.

**Methods:**

A random sample of 799 Dutch employees (response rate 39.6%) completed an internet survey, which comprised two parts. One part of the questionnaire focused on the determinants of cycling behavior including equal numbers of personal, social factors and environmental factors. The other component focused on assessing data on physical activity (PA) behavior. Descriptive and logistic regression analyses were used to analyze factors associated with commuter cycling.

**Results:**

Meeting the physical activity guideline was positively associated with commuter cycling. Television viewing and working full-time were negatively associated. Twenty-six percent of the participants met the PA guideline simply by cycling to work, with health as the main reason. The main barriers for non-cyclists (60%) were perspiration when arriving at work, weather and travelling time. Shorter travelling times compared with other transportation modes were an important facilitator. Environmental factors were positively related to more frequent and more convenient commuter cycling, but they were hardly mentioned by non-cyclists.

**Conclusions:**

This study shows that a relatively large group fulfils the PA recommendations merely by cycling to work. Personal factors (i.e., perceived time and distance) are major barriers to commuter cycling and should be targeted in cycling campaigns, especially in subgroups living within cycling distance to work. Targeting environmental determinants in such campaigns seems to be less important in the Netherlands.

## Background

Daily cycling to work has been shown to improve physical performance and health in men and women [1,2]. It is very common in the Netherlands: the most recent data show that one quarter of commuting journeys are by bicycle [3]. However, despite the effort going into campaigns to promote commuter cycling, about 30% of commuter journeys up to 5 kilometers are still by car.

Furthermore, in recent years an attractive built environment has been emphasized as an important factor in stimulating physical activity (PA). Several studies point at the importance of the built environment for active travel [4-9]. However, most of these studies did not particularly focus on cycling (to work) separately and were conducted in the United States and Australia, where the environment is not as cycle friendly as in the Netherlands.

The question is how to stimulate commuter cycling more effectively. This article aims to contribute to a better understanding of the perceived barriers and facilitators of cyclists/non-cyclists and personal factors associated with commuter cycling.

## Methods

A Dutch internet panel was used (N = 101,000, aged 13-65). In the spring of 2008, a random representative sample of participants from this panel (N = 2014, aged 18-64) were approached to complete the internet survey. Valid responses of 945 participants were obtained. 799 participants were eventually included in the analyses, after excluding participants with a chronic illness that interfered with their PA from the analyses (15.4%).

As well as collecting information about the population demographics, the web-based survey comprised two main parts. One part of the questionnaire focused on the determinants of cycling behavior. Participants were asked separate questions about their rationale for cycling to work (or not) ('*why do (don't) you cycle to work*?'). In addition, questions were asked about facilitators and barriers ('*what could be improved so that you would cycle to work more often or start cycling to work?*). Each question could be answered by selecting the three main reasons or facilitators/barriers from a predetermined list of at least 13 options per question. The lists included equal numbers of personal, social factors and environmental factors.

The other component focused on physical activity (PA) behavior, using the validated Short Questionnaire to Assess Health-Enhancing PA (SQUASH) [10]. This questionnaire covers 14 specific PA behaviors, including cycling and walking to work. These data were used to calculate the percentage of participants meeting the moderate- and vigorous-intensity PA guideline [11]. In addition, sedentary behavior (hours/weeks spent on desk work and television viewing) were included in the survey. Television viewing time was dichotomized as low (<14 hours/week) and high (≥14 hours/week) in line with the study by Salmon et al. [12].

### Analyses

Descriptive analyses were used. In addition, univariate and multivariate analyses were performed using logistic regression to identify the factors associated with cycling to work. Backward stepwise regression was used for the multivariate analyses. It started with a full or saturated model and non-significant variables were iteratively eliminated from the model. The fit of the model was tested after the elimination of each variable to ensure that it still fitted the data adequately. Sociodemographic variables such as gender, age, income and education were examined as possible effect modifiers or confounders. SPSS version 14.0 was used for the analysis and p-values < 0.05 were considered significant.

## Results

### Sample characteristics

Valid responses were obtained from 799 participants (39.6% response rate) with a mean age of 41 years (50% female) (Table 1). About 40% (n = 323) of the sample cycled to work at least once a week and 32% were regular cyclists (≥ 3 times/week). The majority (71%) lived no more than eight kilometers from work. The average single trip distance to work was 6.0 kilometers (median 5 km). Women cycled significantly fewer kilometers (5.3 km) to work than men (6.3 km). About 10% of the cyclists lived more than 16 kilometers from work, with an average of 5 kilometers being travelled by bicycle (i.e. bike and ride). Sixty-five percent of the cyclists met the moderate-intensity PA guideline and 26% of the participants met the PA guideline merely by cycling to work (significant gender difference: men 37% and women 18%; p < 0.001). A significantly smaller (p < 0.001) proportion of the non-cyclists (41%) met the moderate-intensity PA guideline and 29% lived within cycling distance from work (i.e. ≤ 8 km).

**Table 1:** Characteristics of cyclists, non-cyclists and total group (% or mean (SD), the Netherlands.

	Cyclists (N = 323)	Non-cyclists (N = 476)	Total (N = 799)
Female (%)	57.9*	43.9	49.6
Mean age (years)	40.7 (10.8)	41.6 (11.2)	41.2 (11.0)
Full timer (≥36 hours/week) (%)	45.8*	66.0	57.8
College education (%)	37.0	32.1	34.0
High Income (≥€35.000 annually) (%)	51.8	56.4	45.4
Living distance to work (%):			
- 0-4 kilometer	35.2	16.6	23.7
- 5-8 kilometer	36.0	12.2	21.3
- 9-12 kilometer	14.7	11.8	12.9
- 13-16 kilometer	3.3	10.3	7.6
- >16 kilometer	10.8	49.1	34.5
Mean cycling distance (kilometers) to work	6.0 (4.4)	N/A	N/A
Positive intention (%)^a ^	N/A	16.4	N/A
Cycling history (% cycling to work for >2 years)	67.5	N/A	N/A
**Physical activity**			
Recreational cycling (%)	27.2	24.8	25.8
Engaging in sport activity (≥1 time/wk) (%)	50.8*	41.4	45.2
Meeting moderate-intensity physical activity guideline (%)^b^	64.7*	40.5	50.3
Meeting vigorous-intensity physical activity guideline (%)^c^	34.7*	18.3	24.9
Meeting physical activity guideline due to cycling to work (%)	25.8	N/A	N/A
**Sedentary behavior**			
Inactive (desk) job (%)	43.5	47.6	46.0
Television viewing time (≥14 hours/week) (%)	53.7*	61.6	58.6

^a ^Do you intend to cycle to work in the next month ('yes' and 'yes, probably')?, **^b ^**Physical activity of at least moderate-intensity for at least 30 minutes on at least five days/week, ^c ^Vigorous-intensity aerobic physical activity for at least 20 minutes three days/week, * Significant difference between cyclists and non-cyclists (p < 0.05)

### Factors associated with cycling to work

Table 2 shows the odds ratios for factors associated with cycling to work. Participants who cycle to work were less likely to work full-time and less likely to watch more than 14 hours of television per week. They were more likely to live closer to work and to meet both the PA guideline as well as the fitness guideline. Age, income, gender, sedentary desk job or being a recreational cyclist were not associated with cycling to work in the multivariate analyses.

**Table 2:** Uni- and multivariate associations with cycling to work (OR, 95%CI and p-value)

		Univariate			Multivariate	
	**OR**	**95%CI**	**p-value**	**OR**	**95%CI**	**p-value**

Female	1.7	1.3 to 2.3	< 0.001	-	-	-
Age (years)	1.0	1.0 to 1.0	0.61	-	-	-
Full timer (< or ≥ 36 hours/week) (%)	0.5	0.3 to 0.6	< 0.001	0.5	0.4 to 0.7	< 0.001
College education	1.2	0.9 to 1.7	0.16	-	-	-
Income (< or ≥ €35.000 annually)	0.8	0.6 to 1.1	0.25	-	-	-
Living distance to work (≤ 8 kilometer)	6.0	4.3 to 8.0	< 0.001	5.5	4.0 to 7.8	< 0.001
Recreational cycling	1.1	0.9 to 1.6	0.34	-	-	-
Engaging in sport activity (≥ 1 time/week)	1.5	1.1 to 1.9	0.009	-	-	-
Meeting moderate-intensity physical activity guideline	2.7	2.0 to 3.4	< 0.001	3.0	2.1 to 4.0	< 0.001
Meeting vigorous-intensity physical activity guideline	2.3	1.7 to 3.2	< 0.001	1.8	1.3 to 2.8	0.002
Inactive (desk) job	0.8	0.7 to 1.1	0.21	-	-	-
Television viewing time (≥ 14 hours/week)	0.7	0.5 to 0.9	0.01	0.6	0.4 to 0.8	0.001

OR = odds ratio, CI = confidence interval

### Rationale and perceived barriers/facilitators

The main aspects of cycling to work were 'living close to work' (54%), 'health benefits' (54%) and 'getting enough exercise' (31%). Environmental aspects, such as paid parking (5%) and accessibility of the working location (4%), were not considered important about their physical environment. Forty-nine percent of the cyclists thought nothing could be improved. Otherwise, the facilitators mentioned most often were 'better or more cycle paths' (16%), 'a company bicycle' (13%) and 'more support from the employer' (bicycle maintenance, mileage allowance; both 11%). Environmental facilitators were: less delay due to traffic (12%), attractive routes (11%), cycling together (7%) and better facilities (cycle racks, showers and dressing rooms; 7%).

Besides the obvious reason ('living too far from work'; 41%), the three main reasons stated by non-cyclists for not cycling to work were 'sweating' (30%), 'weather dependency' (23%) and 'too time-consuming' (23%). Alongside 'living closer to work' (39%), the three main facilitators for starting to cycle to work were 'shorter travelling time compared with other means of transportation' (20%), 'cycling together' (14%) and 'if I didn't need my car that much for my job' (10%). Only relatively few non-cyclists stated environmental factors (i.e. paid parking, better/more cycling paths, better facilities at work etc.) and employee support as facilitators of commuter cycling (all ≤ 6%). Finally, 22% stated that they would never cycle to work.

## Discussion

The goal of this study was to obtain information about perceived barriers and facilitators among cyclists and non-cyclists with the aim of encouraging commuter cycling more effectively.

Before discussing these results, it should be mentioned that our average cycling distance to work was about 6.0 km, which is higher than the average cycling distance in the Netherlands (4.3 km) [3]. This can be explained in part by the relatively large proportion (10%) of cyclists living more than 16 km from work. Their average cycling distance was 5 km, which implies that they combine public transport and cycling (bike and ride). The national figure includes only the main mode of transport, so the higher average cycling distance in our study could be more representative for actual commuter cycling. Results also indicated that one quarter of the participants met the PA recommendations only by cycling to work. Furthermore, our results suggest that commuter cycling supplements other modes of exercise, and is not a substitute for them. It can be concluded that commuter cycling makes an important contribution to meeting the PA recommendations in the Netherlands.

Health was the most commonly stated reason for cycling to work. In contrast to current scientific interest in health-environment interaction [4-9,13], reasons with an environmental context (paid parking, not being able to park, accessibility of the work location) were not considered important. A partial explanation here is that the Dutch cycling infrastructures and facilities are already good compared with other countries. On the other hand, environmental improvements (e.g. more/better cycling paths) were regarded as important facilitators of more frequent and/or more convenient commuter cycling. Nonetheless, a large proportion of the cyclists thought nothing could be improved.

The non-cyclists did not see the environment as an important facilitator. Perceived barriers for this group were mainly personal, such as the perceived intensity of cycling (and perspiration as a consequence), bad weather and the time needed. These findings concur with an Austrian study [14] and a Belgian study [15]. The latter suggested that, when people live in a setting with an adequate bicycle infrastructure (like the Netherlands), individual determinants outperform the role of environmental determinants. Interventions and campaigns encouraging cycling to work in the Netherlands may therefore have to focus more on personal factors than on changing the environment.

Non-cyclists also often mentioned the perceived time needed for cycling. It has been found that non-cyclists think the time needed to cycle to work is significantly longer than cyclists [15]. Non-cyclists also often lack information about the fastest and most convenient cycling routes. Moreover, research shows that non-cyclists perceive a relatively small riding distance to be too far, while the same distance is covered by the majority of the cyclists on a regular basis [16].

Judging by the relative large proportion of non-cyclists who live within cycling distance to work (< 8 km), there is much to gain when targeting this subgroup; the reduction of (perceived and actual) travelling time, should be one of the main components of future interventions and campaigns for this subgroup. This could be achieved by drawing the attention of non-cyclists to specific planning tools which provide factual information on available and efficient (including travel time) bike routes to their worksite. Communicating the advantages of combining public transportation with cycling to and from the station might also be important, as it is an effective way to shorten travel time and to get enough weekly exercise at the same time.

The most important limitation of this study is the fact that a 'rate of agreement' answer was not used for every separate reason stated in the questions. This method would have allowed us to use the results from this part of the questionnaire in the regression model for this study. The internet questionnaire in this study already covered several topics and using this method would have made completion of the questionnaire too time-consuming, compromising the response rate and the quality of the answers. However, we do acknowledge that our approach may have resulted in only a coarse picture of the reasons and facilitators for commuter cycling. More in-depth qualitative research may be needed to verify these findings and to examine the background to them.

## Conclusions

Cycling to work is a major component of exercise in the Netherlands, allowing a relatively large group to fulfill the PA recommendations. Personal factors (i.e., perceived time and distance) are major barriers to commuter cycling and should be targeted in cycling campaigns, especially in subgroups living within cycling distance to work. Targeting environmental determinants in such campaigns seems to be less important in the Netherlands.

## Competing interests

The authors declare that they have no competing interests.

## Authors' contributions

LHE has made a substantial contribution to conception and design, collection of the data, analysis and interpretation of data. He wrote sections of the manuscript and provided critical revisions for important intellectual content.

IJMH has made a substantial contribution to conception and design written sections of the manuscript and was responsible for revising it critically for important intellectual content. Both authors have given final approval of this version to be published.
